# Normothermic Machine Perfusion (NMP) of the Liver as a Platform for Therapeutic Interventions during Ex-Vivo Liver Preservation: A Review

**DOI:** 10.3390/jcm9041046

**Published:** 2020-04-07

**Authors:** Fungai Dengu, Syed Hussain Abbas, Georg Ebeling, David Nasralla

**Affiliations:** 1 Oxford Transplant Centre, Nuffield Department of Surgical Sciences, University of Oxford, Oxford OX1 2JD, UK; hussain.abbas@nds.ox.ac.uk (S.H.A.); georg.ebeling@nds.ox.ac.uk (G.E.); david.nasralla@nds.ox.ac.uk (D.N.); 2 Department of Hepatopancreatobiliary and Liver Transplant Surgery, Royal Free Hospital, Pond St, Hampstead, London NW3 2QG, UK

**Keywords:** normothermic machine perfusion, therapeutics, liver transplantation, immunomodulation, ischaemia reperfusion injury, microcirculation, organ reconditioning, steatosis, de-fatting

## Abstract

Liver transplantation is increasingly dependent on the use of extended criteria donors (ECD) to increase the organ donor pool and address rising demand. This has necessitated the adoption of innovative technologies and strategies to protect these higher-risk grafts from the deleterious effects of traditional preservation and ischaemia reperfusion injury (IRI). The advent of normothermic machine perfusion (NMP) and rapid growth in the clinical adoption of this technology has accelerated efforts to utilise NMP as a platform for therapeutic intervention to optimise donor livers. In this review we will explore the emerging preclinical data related to ameliorating the effects of IRI, protecting the microcirculation and reducing the immunogenicity of donor organs during NMP. Exploiting the window of opportunity afforded by NMP, whereby the liver can be continuously supported and functionally assessed while therapies are directly delivered during the preservation period, has clear logistical and theoretical advantages over current preservation methods. The clinical translation of many of the therapeutic agents and strategies we will describe is becoming more feasible with widespread adaptation of NMP devices and rapid advances in molecular biology and gene therapy, which have substantially improved the performance of these agents. The delivery of novel therapeutics during NMP represents one of the new frontiers in transplantation research and offers real potential for successfully tackling fundamental challenges in transplantation such as IRI.

## 1. Introduction

Liver transplantation is the only successful treatment option for many patients with end-stage liver disease. With liver disease-related mortality rising globally [1], the demand for suitable organs for transplantation is set to continue to increase. Conventionally, donor livers are preserved at 4 °C in static cold storage (SCS) prior to implantation, but if prolonged, this can be harmful, particularly if the organ is of marginal quality or deemed ‘high-risk’ owing to donor characteristics. These extended criteria donor (ECD) livers typically come from older, higher body mass index (BMI), medically co-morbid donors and donors after circulatory death (DCD). Consequently, the livers are at increased risk of primary non-function (PNF), early allograft dysfunction (EAD) and post-reperfusion syndrome (PRS), and are associated with greater complications such as non-anastomotic biliary strictures and ischaemic cholangiopathy [2,3]. The underlying mechanism or pathological process driving these complications is the increased susceptibility of ECD livers to severe ischaemia-reperfusion injury (IRI) [4,5] which is itself exacerbated by prolonged cold storage. As such, ECDs have been limited in use to situations where preservation times, in particular cold ischaemia times (CIT), are very short and no other suitable donor options exist [6,7,8].

## 2. Normothermic Machine Perfusion of the Liver

Alternative preservation strategies have been developed for livers, lungs, hearts and kidneys to minimise the deleterious effects of SCS and institute protective measures during preservation using machine perfusion [9,10,11,12]. In liver transplantation, these technologies have rapidly expanded with multiple devices and systems routinely being used clinically. Normothermic machine perfusion (NMP) has proven to be an exciting technology, with international multicentre randomized control trial data demonstrating its ability to reduce graft injury, prolong preservation and increase utilisation [13,14]. Further studies have reported on sequential SCS and NMP with comparable clinical outcomes to continuous NMP alone, providing robust clinical data in support of the more logistically straightforward ‘back to base model’ of liver NMP [15]. Although improved utilisation has been demonstrated and viability assessment protocols successfully established (by the Cambridge and Birmingham UK Groups) in ECD livers, NMP alone may not be sufficient to improve clinical outcomes [16,17]. As a result, multiple groups are now working on the use of NMP as a platform for delivering novel therapeutics to recondition, repair and optimise organs for transplant. In a recent landmark study by the Zürich group, Eshmuminov and colleagues were able to normothermically preserve initially porcine, then human, discarded livers for 7 days ex-situ, maintaining physiological parameters [18]. Although this study did not have a transplant recovery model and the discarded human livers were heterogenous therefore impacting the outcomes, the establishment of prolonged preservation is potentially transformative for the field, particularly in the area of therapeutic delivery during liver NMP.

The pre-clinical studies that have provided much of the evidence reviewed in this article are predominantly animal models and discarded human liver studies, which, although they provide excellent experimental models with a strong track record of clinical translation, do have several weaknesses. Animal models of ex-vivo perfusion are not exempt from the drawbacks of other animal studies, including genetic, physiological and immunological differences from human organs, limiting our ability to extrapolate results. Furthermore, transplantation animal models often require more animals, recovery of animals and carry the risk of procedure-related complications, therefore coming into conflict with the 3Rs (Replacement, Reduction, and Refinement) principles of preclinical research [19], when in some cases this can be avoided. To overcome these concerns, some groups have focused on discarded human livers, typically procured with the intent of transplantation but deemed not suitable for clinical use [20]. These organs provide valuable insights but vary dramatically in key characteristics (e.g., preservation times, malignancy, fibrosis and steatosis), many of which can directly impact the parameters being investigated. As a result, a combination of large animal transplant models and discarded human liver studies is increasingly being adopted as the optimal translational approach [18].

Transplantation is unique in that to some extent clinicians have precise timings for key stages of the process, such as donation (cold flush/cross-clamp), preservation, reperfusion and timing of donor graft exposure to the recipient immune system. This permits accurate prediction of re-infection/transfection, allorecognition and tumour seeding; thus, the modification of graft during preservation or in anticipation of these events has become logistically feasible and may dramatically alter the outcomes for transplant recipients.

In this review, we explore the role of NMP of the liver in the delivery of therapeutic agents with the ultimate aim of improving transplant outcomes.

## 3. Ischaemia Reperfusion Injury

Ischaemia reperfusion injury (IRI) remains a necessary component of solid organ transplantation and involves a surge in pro-inflammatory cytokines such as interleukin-1 (IL-1) & interleukin-6 (IL-6), tumour necrosis factor (TNF-α), increased reactive oxygen species (ROS), neutrophil infiltration and complement activation in response to the reperfusion of a previously ischaemic organ, ultimately resulting in apoptosis and necrosis [21,22]. Developing methods to mitigate these effects of IRI have been the subject of intense investigation in the field of transplantation for decades, as the severity of this process has been associated with higher discard rates of organs.

### Gene Silencing with RNAi

Novel agents are increasingly being used to target the pathways of IRI including RNA interference (RNAi). This naturally-occurring mechanism for gene expression downregulation by specific targeting of messenger RNA (mRNA) transcripts [23] is being utilised in the context of liver transplantation to influence IRI-related pathways. Since the discovery of RNAi in 1998, it has opened up new opportunities for specific therapeutic targeting of genes involved in processes of interest and was subject the Nobel Prize in Medicine in 2006 owing to the multitude of potential applications. Pre-clinical work in liver transplantation has provided proof of concept, with Li et al. 2007 using small interfering RNA (siRNA) targeting the Fas gene and Wu et al. 2016 using short hairpin RNA (shRNA) to target interleukin-1 receptor-associated kinase-4 (IRAK 4) in rat models of liver transplantation, demonstrating improvements in markers of hepatocellular damage associated with reduced pro-inflammatory signalling via the interference of the specifically targeted pathways in both studies [24,25]. However, the successful translation of RNAi to clinical transplantation has been limited by costs, perceived risks of delivering the medication systemically and chemical structural challenges related to uptake, stability and selectivity of the therapy. Many of these hurdles are being overcome by a number of different technical advances comprehensively outlined in a recent review by Thijsesen et al., 2019 [26]. The same authors have also described the innovative lipid-based nanoparticle delivery of siRNA to genetically target livers during ex-vivo NMP in a rat model. They have been able to silence the antiapoptotic gene p53 in an in-vivo IRI model of clamping the hepatic inflow as well as demonstrate the feasibility of utilising NMP as a platform for delivering their lipid nanoparticle loaded with siRNA [27]. Interventions described in this review are predicated on normothermia; however, interesting work in this therapeutic class and others has yielded results suggesting that normothermic delivery may not have an effect on the ability to deliver these therapies, opening the possibility of incorporating these therapies into standard cold organ preservation solutions [28]. Beal and colleagues have also explored the effects of delivering an opioid agonist during NMP in a small animal model and found evidence of protection against hypoxic stress and improved mitochondrial function (Table 1).

## 4. Immunomodulation

Following the Consortium for Organ Preservation in Europe (COPE) international clinical trial of NMP for liver transplantation, we found that immune-mediated processes were found to be positively impacted by NMP (e.g., reduced PRS in NMP cohort) corroborating pre-clinical work previously conducted within the group [12,14,31]. Furthermore, subsequent clinical studies exploring the use of NMP following longer periods of cold preservation in both North America and Europe have shown favourable outcomes for NMP with respect to leucocytosis and inflammatory cell infiltrates histologically [15,32]. Mechanistically, this has been explored by Jassem et al. (2018), where the group found that the proportion of tissue-resident CD4^pos^CD25^high^CD127^neg^FOXP3^pos^ regulatory T cells (Treg) seems to increase through the course of NMP, with more interferon-gamma (IFN-γ) and interleukin 17-producing T cells present and less necrosis and apoptosis in the liver parenchyma, associated with less neutrophil infiltration compared to SCS livers [33]. The immune effects of NMP observed through clinical measures such as PRS in the aforementioned clinical studies must, however, be interpreted carefully, as immunophenotyping and advance immune cell analysis of the samples is yet to be reported.

### 4.1. Cell Therapy during NMP

Cellular therapies (e.g., regulatory T cells, tolerogenic dendritic cells, mesenchymal stem cells) and cell-derived products (e.g., extracellular vesicles) are used in organ transplantation with immunomodulatory intent and typically delivered systemically with a well-established safety record [34,35,36]. In liver transplantation, Todo et al. 2016 published the results of a pilot that have accelerated the rate of progress in the field, where 7/10 of the patients treated with a regulatory T-cell (Treg)-based cellular therapy with tolerogenic intent were successfully weaned off all immunosuppression following living donor liver transplant (LDLT), with 16–33 months of rejection-free graft survival [37]. However, challenges remain: the delivery of these cells to the target organ remains problematic, reproducibility is a concern owing to the lack of standardisation of manufacturing processes, the paucity of good manufacturing practice (GMP) facilities and prohibitive costs are all holding back progress. Furthermore, they are largely limited to the living donor pool of recipients, which in the majority of Europe and North America remains a small proportion of overall liver transplant activity [38]. The intrinsic benefits of NMP in cellular therapy delivery are therefore intuitive; the ex-vivo circuit offers the opportunity to directly deliver cells to the liver, using lower doses (cell numbers) will reduce costs and expand therapies to deceased donor receipts. Furthermore, ECDs form a substantial and increasing portion of deceased donor organs and therefore stand to benefit most from these interventions and therapies.

### 4.2. Extra-Cellular Vesicles (EVs) during NMP

Mesenchymal stem cells (MSCs) are immunomodulatory cells that have received widespread attention owing to their purported capacity to alter immune responses favourably in the context of solid organ transplantation [39,40]. Their therapeutic function occurs via paracrine effects related to extracellular vesicles (MSC-EVs) released by the cells. The MSC-EVs are small vesicles released by MSCs that deliver small proteins, microRNA (miRNA), mRNA and even DNA, which exert an effect on target tissues [41]. In the lung, where the majority of the pre-clinical data exists for the use of MSC-EVs, investigators have been able to provide data supporting their efficacy in rodent IRI models. Stone and colleagues assessed the effect of MSC-EVs under both in-situ and ex-situ IRI conditions, yielding mechanistic information on the reduction of pro-inflammatory cytokines and an increase in protective cytokines [42]. This is in keeping with the findings of Lonati and colleagues, who were able to ameliorate the effects of IRI in rat lungs and demonstrate an significant alteration in gene expression related to EV treatment [43]. Overall, a limited number of studies have reported on the use of MSC-EVs during machine perfusion in the various organ systems [42,44]; however, the first group to deliver these cells or cell-derived products to livers during NMP MSC-EVs was Rigo et al. The authors used a rat liver NMP model, perfusing grafts for 4 h and delivering human liver stem-like cell (HLSC)-derived EVs, and assessed uptake within the liver and their effect on hypoxic tissue injury (the latter was induced by suboptimal oxygenation during NMP). They were able to show reduced hepatocellular damage histologically as well as reduced markers of hepatic cytolysis in the NMP perfusate while demonstrating engraftment of the delivered cell product. A limitation of this study was the lack of precision associated with “suboptimal oxygenation” during NMP, which therefore reduced the study’s validity as a model of IRI. Recently, Dondossola and colleagues have addressed this by describing a model of rat liver NMP using washed human red blood cells as an oxygen carrier to maintain optimal oxygenation during NMP and allow direct evaluation of IRI interventions during rat liver NMP in a controlled manner [45]. Although promising, the clinical application of EVs faces numerous challenges, including the standardization of isolation, qualification, characterisation and large-scale clinical-grade production of EVs, as is the case for many novel cell-based therapies. 

### 4.3. Tolerance 

Immunomodulation of grafts during NMP for transplant tolerance is an emerging area of interest, with many groups, including our own, working on this specific area. Ling Yee Chin et al. 2019 reported on a set of experiments where, during a 6 h rat liver perfusion model, they delivered a genetically engineered rat fibroblast cell suspension. They were able to engraft these cells in the donor liver while ensuring the cells were viable and the NMP remained otherwise unaffected [46].

Although progress is being made in this area, many of the studies are limited by the lack of a transplant model and the translational limitations of small animal models, and few studies are able to provide any mechanistic evidence for observed effects.

## 5. Microcirculation and Endothelial Protection

DCD liver transplantation is increasingly common, as with optimised protocols and novel technologies being employed, outcomes have dramatically improved [3,7,8]. These approaches have focused on ways of protecting DCD grafts from the effects of IRI on the microcirculation of the liver, where sinusoidal endothelial cells (SEC) are particularly vulnerable to IRI. During the warm ischaemic phase of DCD procurements, hypo-oxygenation results in rapid depletion of adenosine triphosphate (ATP), mitochondrial stress, activation of Kupffer cells and endothelial dysfunction as SECs undergo necrosis and apoptosis resulting in microcirculatory disturbance [4,5]. NMP has been an effective strategy in mitigating some of these effects, with extensive pre-clinical [47,48] and clinical work [17,49] demonstrating its benefits. Goldarecena et al. have sought to investigate approaches that may demonstrate additional benefit beyond NMP through the delivery of a combination of anti-inflammatory agents and other strategies employable during machine perfusion. They used a porcine liver transplant model, comparing NMP alone with a sub-normothermic machine perfusion (33 °C) intervention group within which the anti-inflammatory medications were delivered (Table 2) [50]. Although the integration of multiple combinations of interventions made it difficult to determine which agents were the most effective and in which combination, it proved some evidence that these agents could ameliorate the effects of IRI and therefore have a role in therapeutic interventions during NMP.

## 6. Microbial Transmission 

The process of transplantation carries a risk of microbial transmission from donor to recipient and re-infection of graft following reperfusion [52]. NMP may provide an opportunity to minimise this. The use of antibiotics during NMP of the liver is well established and has been a part of our group’s clinical protocol [12]; however, this not true of all organs, for example, the kidney [11], where no antimicrobial therapies given during perfusion and infective complications have been reported [53].

Hepatitis C Virus (HCV) has previously been the leading indication in North America for liver transplantation until very recently, having been surpassed by alcohol-related liver disease (ARLD) and non-alcoholic fatty liver disease (NAFLD) due to the advent of improved treatments for HCV. It remains an important condition globally for the development of end-stage liver disease and the consequent requirement for liver transplantation [54]. HCV is associated with high re-infection of the graft following reperfusion and the development of cirrhosis due to aggressive recurrence of HCV, and even in the era of direct-acting antiviral agents (DAAs), there is no consensus on how best to avoid it [55]. The Toronto group have investigated the use of Miravirsen in the induction of resistance to re-infection of livers for transplant during NMP. Miravirsen or antisense miRNA is a locked nucleic acid antisense oligonucleotide which inactivated miRNA-122, a critical factor in the hepatic replication of HCV. In this proof of concept study, Miravirsen was delivered to porcine livers during NMP vs. SCS, and they were able to demonstrate increased functional miRNA-122 sequestration, higher target gene suppression and enhanced absorption [56]. Although this was a key study in the development of NMP as a platform for therapeutic intervention, there were some limitations, including the lack of a porcine HCV model, resulting in an inability to directly assess the efficacy of the intervention and limiting the generalisability of the findings.

## 7. Hepatic Steatosis and Implications on Liver Transplantation

In efforts to increase the donor pool, more “marginal” donor livers are being transplanted [57]. These include livers with substantial intra-cellular fat deposition (steatosis). Steatosis results from altered metabolism of fatty acids within hepatocytes and is characterised by intracytoplasmic accumulation of triacylglycerol (TG) in the form of lipid droplets (LDs) [58]. Large intracystoplasmic LDs cause peripheral displacement of the cell nucleus resulting in macrovesicular steatosis (MaS). Livers with evidence of MaS are much more susceptible to ischaemia reperfusion injury (IRI) during conventional cold storage of the organ, termed static cold storage (SCS). The consequent organ injury is attributed to impaired microcirculation, reduced mitochondrial function and excessive inflammatory response, and is associated with subsequent poor post-transplant outcomes [59].

A recent systematic review concluded that livers with moderate to severe steatosis (more than 30% MaS) are associated with primary non-function and increased graft loss of 71% (RR 1.71, *p* = 0.007), and such high-risk organs are frequently declined for transplantation [60]. As a result, 1000 steatotic livers are retrieved but discarded each year in the USA [61]; in the UK, 39% of liver discards are due to steatosis [62]. 

The incidence of hepatic steatosis, which is commonly associated with obesity, is increasing and currently affects 33% of the UK population [63,64]. Increasing obesity in the population is reflected in deceased organ donors; those with a BMI > 30 kg/m^2^ have increased from 16% to 26% in the last decade [64]. Steatosis in donor livers is inevitably increasing, and methods to render these organs suitable for transplantation are urgently needed. Recently, the benefits of NMP have been described (Table 3) [14,17,65,66].

### 7.1. NMP, Hepatic Steatosis and Pre-Clinical Animal Studies

Steatotic livers constitute the largest individual cohort of organs which might benefit from active intervention during NMP [67,68,69,70,71]. Pre-clinical models demonstrate that ex-situ liver function can be enhanced by NMP, and hepatic triglyceride content can be reduced by enhancing intracellular lipid metabolism and potentiating the reversal of steatosis [67,68].

Jamieson et al. [67] investigated the effect of NMP alone on steatotic porcine livers during 48 h perfusions. Hepatic steatosis was induced by pre-treatment with streptozotocin together with a high fat diet to facilitate hyperglycaemia and a ketotic state prior to organ procurement for the NMP experiments. This study demonstrated that steatotic porcine livers were able to maintain perfusate base excess, factor V and bile production during NMP. In addition, the steatotic livers demonstrated comparable haemodynamics and markers of liver injury to lean controls. Importantly, these livers had higher perfusate glucose and urea levels. Overall, there was a reduction in MaS from 28% to 15% with reduction in lipid droplet size at the end of preservation without any defatting adjunct therapy.

Nagarth et al. [68] developed an experimental oxygenated normothermic model investigating the effect of a “defatting cocktail” on steatotic livers procured from Zucker rats over 3 h perfusions. The “defatting cocktail” consisted of a combination of pharmacological agents, including the peroxisome proliferator-activated receptor delta (PPARδ) ligand GW501516, the peroxisome proliferator-activated receptor alpha (PPARα) ligand GW7647, the cyclic adenosine monophosphate (cAMP) activator forskolin, the pregnane X receptor ligand hypericin, the insulin-mimetic adipokine visfatin and the constitutive androstane receptor ligand scorparone (see Table 4). In combination with NMP, there was a reduction in hepatic triglyceride content of 65% and intracellular lipid content by 50% with increased hepatic lipid metabolism. However, a 30% reduction in hepatic triglyceride was seen in the control perfusate with no defatting agents.

Raigani et al. [72] demonstrated similar results using an addition of l-carnitine (to increase fatty acid β-oxidation) to the “defatting cocktail”. The overall reduction of MaS was 41.5% to 8.5% following deffating interventions. In addition, there was an increase in perfusate ketone content (a marker of fatty acid β-oxidation), bile bicarbonate content and lactate clearance in steatotic livers that received the interventions.

These pre-clinical animal studies have provided an insight into the potential of NMP as a platform to provide active intervention to treat donor hepatic steatosis. They provide evidence that both hepatic triglyceride content and MaS can be reduced during ex-situ perseveration. However, these studies include a small sample size of homogenous livers in which steatosis has been induced as part of the experimental design. Therefore, there is a risk of translational bias if results are applied to clinical practice in a heterogenous cohort of steatotic human livers procured for transplantation. Overall, the benefits of defatting agents in animal studies appear conflicting; NMP alone has been shown to reduce hepatic triglyceride content with improved liver function comparable with lean counterparts. The effect of defatting agents during NMP would be better investigated in a discarded steatotic human liver model (Table 5). 

### 7.2. NMP, Hepatic Steatosis and Discarded Human Livers

Although animal models have demonstrated the benefit of NMP in hepatic lipid metabolism, a recent study involving NMP alone of human steatotic livers subjected to 24 h perfusions did not demonstrate a reduction in macrosteatosis [73]. However, Ceresa et al. have recently explored the effect of NMP on steatotic human livers and observed clear differences in fat metabolism during preservation compared to lean livers, with greater mobilisation of TG and mitochondrial fatty acid β-oxidation. However, the degree of MaS did not change significantly during NMP when compared to SCS [71]. In a recent trial, previously declined livers were perfused, and those meeting pre-defined functional criteria were transplanted: 22 of 31 perfused organs were transplanted, all with immediate function. Notably, of the livers that did not meet viability criteria, a large proportion had evidence of moderate to severe steatosis and were therefore not transplanted [74]. These data suggest that steatotic human livers require active intervention beyond that of simply replacing SCS with NMP. 

Boteon et al. [69] have demonstrated the potential of a defatting strategy in organs retrieved for clinical transplantation but discarded due to advanced steatosis using a “defatting cocktail” described in pre-clinical animal studies (Table 4) with the addition of l-carnitine during NMP. Using groups of five steatotic human livers, pharmacological defatting was associated with improved metabolic function, reduced vascular resistance, lower levels of liver injury and increased bile production. There was evidence of reduction in markers of oxidative injury, immune cell activation, release of inflammatory cytokines and tissue triglycerides. There was a reduction of MaS by 40% at 6 h of perfusion (Table 5).

Interestingly, the authors demonstrated that five livers that received defatting therapies were able to achieve viability criteria for transplantation compared to two in the control group (*p* = 0.04). However, not all treated livers that meet the viability criteria achieved MaS of <30% [69]. This would suggest that evidence MaS of <30% following intervention on NMP may not be a necessary criterion to proceed with transplantation. In fact, the results would suggest that organ recovery during NMP could be a separate process to defatting. Thereby, activation of cytoprotective and vasoprotective pathways might be what is required to render such organs suitable for transplantation [75].

Although Boteon et al. [69] demonstrated metrics that would have rendered the organs transplantable, further evaluation of the safety profile of the proposed “defatting cocktail” is required prior to approval for human use and translation of findings. Currently, many of the agents included in the “defatting cocktail” lack important safety data (although there is some cytotoxicity reported in vitro) [76]. Hypericin is a component of St John’s Wort that is involved in up-regulation of the cytochrome P450 3A4 enzyme [77]. This enzyme is involved in the metabolism of medications including cyclosporine and tacrolimus. In addition, the peroxisome proliferator-activated receptor agonists GW501516 and GW7647 have not been tested in human trials and concern has been raised regarding carcinogenesis in preliminary animal studies [78]. 

## 8. Conclusions and Future Perspectives

The recent studies of ex-situ defatting in discarded human livers have demonstrated a “proof of concept”, and if these were translated into clinical practice, it would significantly increase the number of safely transplantable high-risk donor organs. Overall, further research would provide a platform for therapeutic ex-situ organ optimisation with novel agents to increase organ utilisation, reduce waiting list deaths and address implications of the global obesity crisis in the context of liver transplantation.

**Figure 1:**
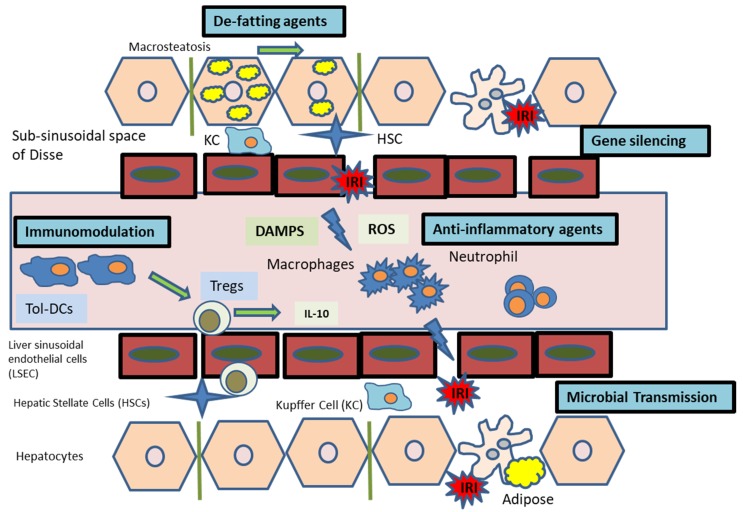
Overview of interventions during NMP for IRI.

We have reviewed the rapidly evolving field of therapeutic delivery during normothermic machine perfusion for liver transplantation (Figure 1). Technological advances in perfusion devices coupled with advances in molecular medicine and development of novel therapeutics have propelled this to the forefront of transplant research. To date, the research has focused on pre-clinical perfusion models, and we expect to see many of these therapies progress to phase I/II studies as acceptance increases.

## Figures and Tables

**Table 1:** Ischaemia Reperfusion Injury.

Study	Model	N	Preservation Details/Groups	Length of NMP	Therapeutic Agent(s)	Outcome
Gillooly et al. 2019 [29]	Rat	N/A	HMP ± siRNA, NMP ± SiRNA,	4 h	Fas-short interfering RNA (siRNA)	Diffuse uptake of siRNA in both NMP and HMP groups, with increased uptake in the latter.
Beal et al. 2019 [30]	Rat	6 per group	NMP, NMP + Enkephalin	4 h	Enkephalin	Reduced hepatocyte oxidative stress and mitochondrial dysfunction via opioid receptor signalling.
Moore et al. 2017 [27]	Rat	N/A	NMP	6 h	p53 siRNA-cy3	Positive fluorescence for cy3 detected in NMP livers.

HMP: Hypothermic Machine Perfusion; siRNA: small interfering ribonucleic acid; Enkephalin: D-Ala2, D-Leu5 (DADLE); cy3-dye.

**Table 2:** Microcirculation protection. HBD: Heart-beating donor; UNHBD: Uncontrolled non-heart-beating donor; SNMP: sub-normothermic machine perfusion.

Study	Model	N	Perfusion Details/Groups	Length of NMP	Therapeutic Agent(s)	Outcome
Hara et al. 2013 [51]	Rat (Reperfusion)	5 per group	HBD (SCS), UNHBD + NMP, UNHBD + NMP +PGE1	30 min	Prostaglandin E1 (PGE1)	Improved mitochondrial function and reduced inflammatory cytokines in NMP + PGE1 group
Goldaracena et al. 2016 [50]	Porcine (Transplant)	5 per group	NMP, SNMP + anti-inflammatory agents, HBD (SCS)	4 h	Anti-inflammatory additives: BQ123, prostaglandin E1, Acetylcystine, prostacycline, gas composition 95% O_2_ and 5% CO_2_	Significantly lower markers of hepatocellular damage in NMP groups, Improved endothelial (microcirulatory) function

**Table 3:** Normothermic machine perfusion (NMP) in liver transplantation.

Key Benefits:
(i) Allows recovery from acute injury (hypoxia) sustained prior to or during retrieval [65];
(ii) Permits objective assessment of organ function prior to transplantation: a number of studies have shown that this enables identification of organs in the ‘high-risk’ category that can safely be transplanted [14,17,66];
(iii) Enables extended preservation times (up to 24 h) [14]
(iv) Provides the opportunity for therapeutic intervention to a functioning organ before it is transplanted.

**Table 4:** Defatting agents.

Defatting Agent	Function
PPARδ ligand GW501516	Increase fatty acid β-oxidation
Peroxisome proliferator-activated receptor (PPAR) α ligand GW7647	Increase mitochondrial fatty acid oxidation
Cyclic adenosine monophosphate (cAMP) activator forskolin	A glucagon mimetic cAMP activator, increases lipolysis and fatty acid oxidation
Pregnane X receptor ligand hypericin	Increase β-oxidation (very long chain fatty acids)
Visfatin	An insulin-memetic adipokine, role not fully understood
Scorparone	An androstane receptor ligand, upregulates PPAR

Defatting agents and mechanism of action in amelioration of hepatic steatosis.

**Table 5:** Summary of defatting interventions and experimental design.

Ref.	Defatting Interventions	Model	Total Ex-Situ Perfusion Time (h)	Percentage (%) Reduction in Macrosteatosis (MaS)	Main Outcomes
Jamieson et al. 2011 [67]	NMP alone	Porcine	48	13	Reduction in hepatic triglyceride content of 31% and markers of hepatocyte injury comparable to lean counterparts
Nagarth et al. 2009 [68]	GW501516, GW7647, forskolin, hypericin, visfatin and scorparone	Zucker rats	3	50	Reduction in hepatic triglyceride content of 65% Increased hepatic lipid metabolism
Raigani et al. 2019 [72]	GW501516, GW7647, forskolin, hypericin, visfatin, scorparone and L-carnitine	Zucker rats	6	33	Hepatic triglyceride content not reported Increased perfusate ketone content, bile bicarbonate content and lactate clearance
Boteon et al. 2019 [69]	GW501516, GW7647, forskolin, hypericin, visfatin, scorparone and L-carnitine	Discarded human livers	6 12	40 50	Reduction in hepatic triglyceride level of 38% at 6 h and 30% at 12 h Increased hepatic lipid metabolism, improved metabolic liver function, reduced vascular resistance and reduced markers of hepatocyte injury Reduced immune cell activation and release of inflammatory cytokines

Outcomes following defatting interventions, including percentage reduction in steatosis, metabolic and synthetic liver function.

## Abbreviations

°C	Degrees Celsius
%	Percent
3R	Reduction, Refinement and Replacement
ARLD	Alcohol-related liver disease
ATP	Adenosine triphosphate
BMI	Body mass index
BQ-123	D-tryptamine-D-aspartic acid-L-proline-D-valine-L-leucine
cAMP	Cyclic adenosine monophosphate
CD4	Cluster of differentiation 4
CD25	Cluster of differentiation 25
CD127	Cluster of differentiation 127
CIT	Cold ischaemia time
CO_2_	Carbon dioxide
COPE	Consortium for Organ Preservation in Europe
Cy3	Cyanine Cy3
DAA	Direct-acting antivirals
DCD	Donors after circulatory death
DNA	Deoxyribonucleic acid
EAD	Early allograft dysfunction
ECD	Extended criteria donor
FOXP3	Forkhead box P3
GMP	Good manufacturing practice
GW7647	2-(4-(2-(1-Cyclohexanebutyl)-3-cyclohexylureido)ethyl)-phenyl-thio)-2-methyl-propionic acid
GW501516	2-[2-Methyl-4-[[[4-methyl-2-[4-(trifluoromethyl)phenyl]-5-thiazolyl]methyl]thio]phenoxy]-acetic acid
h	Hour/hours
HBD	Heart-beating donor
HCV	Hepatitis C virus
HLSC	Human liver stem-like cells
HMP	Hypothermic machine perfusion
IFN-γ	Interferon gamma
IL-1	Interleukin 1
IL-6	Interleukin 6
IL-17	Interleukin 17
IRAK-4	Interleukin-1 receptor-associated kinase 4
IRI	Ischaemia reperfusion injury
kg/m^2^	Kilogram per square meter
LD	Lipid droplet
LDLT	Living donor liver transplant
MaS	Macrovesicular steatosis
mRNA	Messenger RNA
miRNA	MicroRNA
MSC	Mesenchymal stem cells
MSC-EV	Extracellular vesicles of mesenchymal stem cells
NAFLD	Non-alcoholic fatty liver disease
NMP	Normothermic machine perfusion
O_2_	Oxygen
PGE1	Prostaglandin E1
PNF	Primary non-function
PPARα	Peroxisome proliferator-activated receptor alpha
PPARδ	Peroxisome proliferator-activated receptor delta
PRS	Post reperfusion syndrome
RNA	Ribonucleic acid
RNAi	RNA interference
ROS	Reactive oxygen species
SCS	Static cold storage
SEC	Sinusoidal endothelial cell
shRNA	Short hairpin RNA
siRNA	Small interfering RNA
SNMP	Sub-normothermic machine perfusion
TG	Triacylglycerol
TNF-α	Tumour necrosis factor alpha
Treg	Regulatory T cell
UNHBD	Uncontrolled non-heart-beating donor
